# Krill oil supplementation improves transepidermal water loss, hydration and elasticity of the skin in healthy adults: Results from two randomized, double‐blind, placebo‐controlled, dose‐finding pilot studies

**DOI:** 10.1111/jocd.16513

**Published:** 2024-08-21

**Authors:** Katina Handeland, Mike Wakeman, Lena Burri

**Affiliations:** ^1^ Aker BioMarine Human Ingredients AS Lysaker Norway; ^2^ Faculty of Health and Wellbeing University of Sunderland Sunderland UK

**Keywords:** skin, hydration, krill, omega‐3, phospholipids

## Abstract

**Background:**

Dietary marine omega‐3 fatty acids and phospholipids have individually shown favorable effects on skin barrier function. Krill oil offers a combination of omega‐3 in phospholipid form which might enhance the efficacy in supporting skin health.

**Aims:**

The aim was to investigate the impact of two different doses of krill oil on skin transepidermal water loss (TEWL) in healthy adults. Secondary outcomes were skin hydration, elasticity and the omega‐3 index.

**Methods:**

Two randomized, double‐blind, placebo‐controlled, pilot studies were conducted in healthy adults with a baseline TEWL of >10 and ≤24.9 g/m^2^/h. In study 1, 51 participants consumed 1 g of krill oil or placebo daily. In study 2, 50 participants consumed 2 g of krill oil or placebo daily. The outcomes were assessed at baseline, 6 and 12 weeks.

**Results:**

The krill oil supplemented groups significantly increased their omega‐3 index versus placebo in both studies. Furthermore, the krill oil groups in both studies showed statistically significant beneficial reductions in TEWL (from 14.47 ± 3.65 to 13.83 ± 3.78 in study 1 and from 14.25 ± 3.21 to 13.02 ± 2.76 in study 2) and increases in hydration and elasticity when compared to placebo. There were significant linear relationships between changes in the omega‐3 index and changes in TEWL, hydration and elasticity in both studies.

**Conclusions:**

Daily oral supplementation with 1 and 2 g of krill oil showed significant and dose‐dependent improvements in skin TEWL, hydration, and elasticity compared to placebo that correlated with changes in the omega‐3 index.

## 1 INTRODUCTION

In healthy skin, there is a balance between the water content and the amount of water passing through the skin. The skin acts as a protective barrier between the body's internal environment and the external world. Healthy skin is influenced by a complex interplay of many internal and external factors, for example, nutrition, hormones, sun exposure, soap and pollution.^1^

The skin is composed of three main layers: epidermis, dermis, and hypodermis, all three of which vary significantly in their anatomy and function.^2^ The outermost layer of the epidermis, known as the stratum corneum, is recognized to play a crucial role in the skins' barrier function. It prevents excessive water loss through the dermis and avoids that compounds from the environment permeate into the deeper layers and thereby provoke an immune response. The stratum corneum is comprised of cornified keratinocytes (named corneocytes) surrounded by a lipid matrix composed of three main types of lipids: free fatty acids, cholesterol, and ceramides. These lipids play distinct roles in maintaining the structural integrity and functionality of the skin barrier, and alterations in the lipid composition can significantly impact barrier function.^3^

There are three main ways water is lost from the skin's surface: through passive diffusion across skin layers, known as transepidermal water loss (TEWL), through sweat gland excretion, and through the evaporation of water that has been absorbed from the environment.^4^, ^5^ TEWL levels can significantly increase in specific conditions such as dermatitis psoriasis, atopic dermatitis, or different weather conditions and is a standard method for evaluating skin barrier function across various conditions.^6^, ^7^, ^8^ Hence, TEWL is a marker for barrier function, and recognizing standard values can assist in forecasting disease progression in dermatology by quantifying evaporative water loss at the skin's surface.^9^ On the other hand, variables such as age, race, anatomic location, mask‐use, stress, and genetic variability can affect TEWL, making it important to control for these factors to obtain accurate measurements.^10^

Apart from TEWL, another critical parameter for evaluating barrier function is skin hydration,^11^ which refers to the water content within the stratum corneum.^12^ Maintaining adequate hydration in this layer is pivotal for processes such as skin proliferation, differentiation, enzymatic activities, and inflammation.^11^ Age has been identified as a significant factor affecting skin hydration, with older individuals generally exhibiting lower levels.^13^ Water loss and hydration of the stratum corneum are linked to its lipid content and the generation of new lipids in the skin. Both TEWL and skin hydration are influenced by similar factors, showcasing a significant inverse correlation, wherein increased skin hydration is linked to lower TEWL values.^14^

Both long‐chain omega‐3 polyunsaturated fatty acids (LC n‐3 PUFAs) and phospholipids individually have been proposed to contribute to skin health, with their deficiencies resulting in skin dryness, scaling, and increased TEWL.^15^, ^16^, ^17^ As essential membrane components, eicosapentaenoic acid (EPA) and docosahexaenoic acid (DHA) modulate cell membranes and associated enzymes, thereby supporting skin barrier function.^18^, ^19^, ^20^ Additionally, they impact ceramide levels and ceramide fatty acid composition, which are crucial for skin barrier function,^21^, ^22^ and exhibit anti‐inflammatory effects.^23^, ^24^, ^25^ EPA and DHA cannot be produced in the skin and must be obtained from external sources.^26^

Phospholipids, amphiphilic lipids found in all plant and animal cell membranes arranged as lipid bilayers, constitute another dietary component that may beneficially impact the skin.^27^ In hairless mice fed dietary milk phospholipids on top of their standard diet for 6 weeks, stratum corneum hydration was significantly improved, and the amount of ceramides increased compared to the control mice.^28^ Another study in hairless mice reported that the mechanisms behind how milk phospholipids attenuated dry skin conditions, involved modulation of epidermal covalently bound ω‐hydroxy ceramides associated with formation of lamellar structures and skin inflammation.^29^

Krill oil, derived from Antarctic krill (*Euphausia superba*), is a sustainable source of EPA and DHA.^30^ It is different from other n‐3 dietary supplements by that more than 80% of these two fatty acids are bound to phospholipids, predominantly phosphatidylcholine.^31^ This unique combination of EPA and DHA whitin phospholipids suggests a synergistic effect on skin function, potentially benefiting skin health and appearance.

A recent study compared a 12‐week administration of 2 g/day of krill oil daily with placebo in 54 healthy Japanese adults with normal to slightly dry skin. At the end of study, the krill oil group showed significant reductions in TEWL, improved lower eyelid pore size, and a notably increased omega‐3 index, when compared to the placebo group.^32^ Furthermore, two pre‐clinical studies were recently published, looking at the effects of krill oil administration on skin outcomes in mice. Both studies suggest that krill oil positively affects skin health by enhancing it's hydration, collagen, and hyaluronic acid levels while protecting it against UVB‐induced damage and inflammation.^33^, ^34^ Collagen and hyaluronic acid are two key molecules for skin wrinkle formation and skin moisture maintenance,^35^, ^36^ and both of these pre‐clinical studies found that krill oil significantly increased the mRNA expression of hyaluronic acid‐ and collagen‐related genes.

The two pilot studies creating the basis for the results presented here wanted to explore the impact on skin barrier function, hydration and elasticity in a healthy, adult, Western population, and whether there was a dose–response relationship. Hence, the objective of these two separate, dose‐ranging, randomized controlled, double‐blind, pilot studies was to assess whether krill oil administered at a dose of 1 or 2 g per day had a positive effect on non‐invasive measures of skin function, namely TEWL, hydration and elasticity, and whether there were any associations between the omega‐3 index and these skin outcomes.

## 2 METHODS

### 2.1 Trial design

The studies were set up as two separate, double‐blind, randomized, placebo‐controlled, dose‐ranging studies, where the included subjects were randomized to take either 1 g per day of krill oil or the equivalent dose of an identical placebo (study 1), or 2 g per day of krill oil or the equivalent dose of identical placebo capsules (study 2). The studies were conducted between 2016 and 2017 in England. The study duration was approximately 84 days (12 weeks) with an interim study visit after 42 days (6 weeks). In addition, two follow‐up phone calls were conducted after about 22 and 64 days to inquire about adverse events and study compliance. The protocols and informed consent were approved by the Institutional Review Board (IRB) of East Midlands Leicester Central Research Ethics Committee on 15.4.2015 and extended by amendment. The studies were conducted in accordance with the guidelines set forth by the International Conference on Harmonization (ICH) Guidelines for Good Clinical Practice (GCP), and the Declaration of Helsinki regarding the treatment of human subjects in a study.

### 2.2 Participants

For both studies, the participants were recruited via advertisements and leaflets in four local pharmacies. Pharmacy staff were briefed about the study and people interested in participating could discuss their involvement with the staff who also handed out informed consent forms. An appointment was then set up where the chief investigator checked the eligibility criteria by making the skin assessments and collecting blood for the omega‐3 index analysis. The criteria for participation were: healthy individuals aged 18 years or older who were able to give informed consent and with TEWL between >10 and ≤24.9 g/h/m^2^. Participants were excluded from participation if they used anti‐coagulant medication, were allergic to shellfish or were pregnant as determined by a pregnancy test.

The study assumed a large effect size (*d* = 0.80) between the krill oil and the placebo group in terms of change in TEWL at 12 weeks. With a statistical significance level (α) of 5% and statistical power (1‐β) of 80%, the target number of subjects calculated with the statistical program SPSS was 26 subjects per group. However, based on the expectation of potential dropouts, 40 subjects per group were enrolled.

Study participants who met the eligibility criteria during screening were assigned in a 1:1 ratio to either the test product group or the placebo group, using a computer‐generated allocation table created with SPSS and a block random allocation algorithm without stratification. All participants completed a lifestyle and dietary survey upon entering the trial, with a particular emphasis on fish consumption. At each intervention, participants confirmed that these parameters remained unchanged throughout the duration of the study. The dates for inclusion of the first and the last volunteer were April 8, 2017 and September 8, 2017.

### 2.3 Interventions

The study products were capsules that contained 1000 mg Superba Boost™ krill oil or identical equivalent placebo. Each krill oil capsule contained 570 mg/g phospholipids, 170 mg/g EPA and 90 mg/g DHA. The remaining lipid fraction is made up of triglycerides. Each placebo capsule contained a mixed vegetable oil which consisted of a mixture of olive oil (extra virgin, cold pressed), maize oil (refined), palm kernel oil (refined), and medium chain triglycerides, in the ratio 4:4:3:2, this mixture was made to mimic the fatty acid composition in a normal, Western diet. The control and krill oil capsules were identical in look and taste, and both were provided by Aker BioMarine Human Ingredients AS (Lysaker, Norway). The capsules were supplied to the participants at their first study visit. Participants in study 1 took one capsule per day of either krill oil or placebo, participants in study 2 took two capsules per day of either krill oil or placebo.

## 3 Study Outcomes

### 3.1 Transepidermal water loss

Trans epidermal water loss was measured by a Tewameter® TM 300 (Courage + Khazaka electronic GmbH, Cologne, Germany). The tewameter probe measures the density gradient of the water evaporation from the skin of the left inner forearm indirectly by two pairs of sensors (temperature and relative humidity) inside a hollow cylinder, which gives an open chamber measurement. A microprocessor analyses the values. The TEWL measurement is a non‐invasive method commonly used to measure skin barrier integrity, and increased TEWL values are linked to dermatologic diseases such as psoriasis and atopic dermatitis.^37^ TEWL measurements are divided into the categories shown in Table 1

**TABLE 1:** Skin conditions according to TEWL values, as measured by Tewameter® TM 300.

g/h/m^2^	Skin condition
Above 35	Critical
25–34.9	Strained
15–24.9	Normal
10–14.9	Healthy
0–9.9	Very healthy

### 3.2 Elasticity

Elasticity was measured by a Cutometer® MPA 580 (Courage + Khazaka electronic GmbH, Cologne, Germany). The measurement is based on suction. Negative pressure created by a vacuum pump within the device draws the skin into the aperture of the probe. Inside the probe, the penetration depth is determined by a non‐contact optical measuring system. The resistance of the skin to be sucked up by negative pressure (firmness) and its ability to return into its original position (elasticity) are displayed as curves in real time. The measurement with the cutometer is used as standard in anti‐aging research and cosmetology. The elasticity in this trial was given as the portion between the max amplitude and the ability of reformation of the skin, and the closer the value is to 1, the more elastic the curve.

### 3.3 Hydration

Hydration was measured by a Corneometer® CM 825 (Courage + Khazaka electronic GmbH, Cologne, Germany). This instrument is commonly used worldwide to obtain values of the hydration level of the skin surface, mainly the stratum corneum. The measurement is based on capacitance measurement of a dielectric medium. The corneometer measures the change in the dielectric constant due to skin surface hydration by capacitance differences of a precision capacitor. Substances on the skin (e.g., salts or residues of topical applied products) is stated to have minimal influence due to the capacitance measurement, and the measurement depth is very small (10–20 μm of the stratum corneum) to exclude the influence of water in deeper skin layers. An electric field between the tracks with altering attractions develops. One track builds up a surplus of electron (minus charge), the other a lack of electron (plus charge). The scatter field penetrates the very first layer of the skin during the measurement and the capacitance is determined. Measurements are divided into the following categories: normal: 45–130 AU, dry: 30–44.9, very dry: 0–29.9.

### 3.4 Omega‐3 index in red blood cells

The omega‐3 index is defined as the sum of EPA and DHA as the percentage of total fatty acids in erythrocytes.^38^ Analysis of EPA and DHA in erythrocytes was performed with gas chromatography at OmegaQuant in USA. In brief, fatty acid composition is analyzed using HS‐Omega‐3 Index® methodology previously described in reference.^39^ Fatty acid methyl esters are generated by acid transesterification and analyzed by gas chromatography. Fatty acids are identified by comparison with a standard mixture of fatty acids characteristic of erythrocytes and plasma. A total of 26 fatty acids are identified and quantified. For the measurement in erythrocytes, results are given as percentage of total identified fatty acids after response factor correction.

Participants used a test kit with a small, sterile needle to extract a few drops of blood from their fingertip in the morning. After washing their hands, they pierced the skin, collected blood on a test paper, and delivered the kit to the study pharmacy which labeled it with participant ID number and sent it to OmegaQuant' s laboratory for omega‐3 index analysis.

## 4 STATISTICAL METHODS

In order to test for overall and interaction effects of time point (Day 0, Day 42, Day 84; within subjects' factor) and supplement type (Superba krill oil, placebo; between subjects factor) on omega‐3 index, TEWL, Hydration and Elasticity, mixed two‐way ANOVAs were employed separately for each outcome measure.

For post hoc analyses, paired samples *t*‐tests were performed on changes in outcome variables from baseline to study end within each supplementation group (active and placebo) separately. In addition, an independent samples *t*‐test was employed to directly investigate any differences in levels of change between the active group and the placebo group.

All statistical tests were performed two‐sided, and a significance level (*α*) of 0.05 was employed. Bonferroni corrections for multiple comparisons was employed for all post hoc analyses, with *α*/*k* set as the criterion for significance (*k* = number of pairwise comparisons). For the present study, three post hoc tests were performed for each outcome measure, resulting in a significance criterion of 0.05/3 = 0.017.

Effect size estimates were calculated using Cohen's *d*, with values in the range [0.20, 0.49] indicating a small effect size, [0.50, 0.79] indicating a medium effect size, [0.80, 1.19] indicating a large effect size and >1.20 indicating a very large effect size.^40^

Linear regression was employed to examine the relationship between changes in omega‐3 index and changes in TEWL, hydration and elasticity across the study period.

## 5 RESULTS

### 5.1 Participants

**FIGURE 1:**
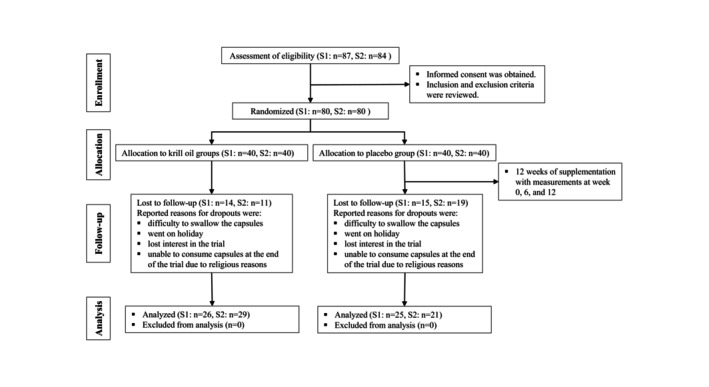
Participant tracking flowchart for Study 1 (S1) and Study 2 (S2).

In study 1, 80 volunteers were assessed eligible and enrolled into the trial (Figure 1). In total, 29 volunteers dropped out, and the reported reasons for the dropouts were (a) difficult to swallow the capsules (this was a significant issue for many lost participants), (b) went on holiday, (c) lost interest in the trial, and (d) unable to consume capsules at the end of the trial due to religious holidays. Fifty‐one volunteers completed the trial, and data from all those that completed were analyzed.

In study 2, also 80 volunteers were assessed eligible and enrolled into the trial (Figure 1). In total, 30 volunteers dropped out, and the reported reasons for the dropouts were similar as for study 1. Fifty volunteers completed the trial, and data from all those that completed were analyzed.

### 5.2 Omega‐3 index

Table 2 shows the omega‐3 index values for volunteers participating in study 1 and study 2 across the three time points (baseline, Day 42, and Day 84). In study 1, the mean omega‐3 index increased from 4.6% at baseline to 5.1% at 42 days and plateaued at 5.01% by 84 days, while the placebo group remained stable at 6.13%–6.05% over time. In study 2, the mean omega‐3 index increased from 5.43% at baseline to 6.80% at 42 days and ended at 6.85% after 84 days, while the placebo group remained stable at 5.51%–5.71% over time. The changes in the omega‐3 index after both doses of krill oil were statistically significant compared to the respective placebo groups in both studies.

**TABLE 2:** Omega‐3 index at baseline, 42 days, and end of trial (84 days). Values are given as mean ± SD.

Group	*N*	Baseline	Day 42	Day 84	Mean change^a^	*p*‐value^b^
Study 1 (1 g/day)						
Krill oil	26	4.57 ± 0.76	5.09 ± 1.03	5.01 ± 0.78	0.44 ± 0.75	0.003
Placebo	25	6.13 ± 1.05	6.01 ± 1.11	6.05 ± 1.03	0.08 ± 0.35	
Study 2 (2 g/day)						
Krill oil	29	5.43 ± 1.02	6.80 ± 1.09	6.85 ± 1.21	1.42 ± 0.98	0.0001
Placebo	21	5.51 ± 1.09	5.71 ± 1.19	5.55 ± 1.03	0.03 ± 0.48	

^a^ Mean ± SD change between baseline and Day 84.

^b^ Independent samples *t*‐test comparing the change in Omega‐3 index recorded for the krill oil and the placebo group between baseline and Day 84.

### 5.3 Transepidermal water loss

In study 1 (1 g/day), the mixed two‐way ANOVA demonstrated a significant interaction effect of time point × Supplement type on TEWL (*p* = 0.001), and this effect was driven primarily by a reduction in TEWL in the krill oil group, not the placebo group. Post hoc analysis confirmed this observation, with a notable decrease in TEWL from 14.47 ± 3.65 g/h/m^2^ (mean ± SD) at baseline to 13.83 ± 3.78 by Day 84 in the krill oil group (*p* < 0.0027; Cohen's *d* = 0.66, corrected for dependence among means) (Table 3). No significant change in TEWL was observed in the placebo group (*p* = 0.315). An independent samples *t*‐test comparing the change in TEWL in the two groups from baseline to Day 84 revealed a significantly greater decrease in TEWL in the krill oil group (*t* = −3.32, *p* = 0.002; 95% confidence interval (CI) = [−1.25, −0.31], Cohen's *d* = 0.96).

**TABLE 3:** Skin TEWL (g/h/m^2^), hydration and elasticity at baseline, Day 42 and end of trial (Day 84). Values are given as mean ± SD.

	Study 1 (1 g/day)					Study 2 (2 g/day)				
	Baseline	Day 42	Day 84	Mean change	*p*‐value^a^	Baseline	Day 42	Day 84	Mean change	*p*‐value^a^
TEWL										
Krill oil	14.47 ± 3.65	14.06 ± 3.58	13.83 ± 3.78	−0.65 ± 0.99	0.002	14.25 ± 3.21	13.47 ± 2.96	13.02 ± 2.76	−1.23 ± 0.73	<0.001
Placebo	13.32 ± 3.63	13.36 ± 4.0	13.46 ± 4.08	0.13 ± 0.64		12.30 ± 2.91	12.13 ± 3.10	12.06 ± 3.10	−0.24 ± 0.41	
Hydration										
Krill oil	46.31 ± 6.61	47.62 ± 6.73	48.31 ± 6.93	2.00 ± 1.67	0.01	47.52 ± 4.42	49.45 ± 4.49	51.17 ± 4.51	3.66 ± 2.14	<0.001
Placebo	41.76 ± 6.43	42.48 ± 6.47	42.56 ± 6.46	0.80 ± 1.50		48.95 ± 7.49	49.19 ± 7.39	49.81 ± 7.66	0.86 ± 1.32	
Elasticity										
Krill oil	0.788 ± 0.080	0.799 ± 0.078	0.803 ± 0.079	0.015 ± 0.010	<0.001	0.830 ± 0.075	0.848 ± 0.075	0.858 ± 0.074	0.028 ± 0.010	<0.001
Placebo	0.860 ± 0.081	0.861 ± 0.082	0.863 ± 0.08	0.003 ± 0.001		0.852 ± 0.085	0.853 ± 0.084	0.856 ± 0.084	0.004 ± 0.010	

^a^ Independent samples *t*‐test comparing the change in the skin health outcomes recorded for the krill oil and the placebo group from baseline to Day 84.

**FIGURE 2:**
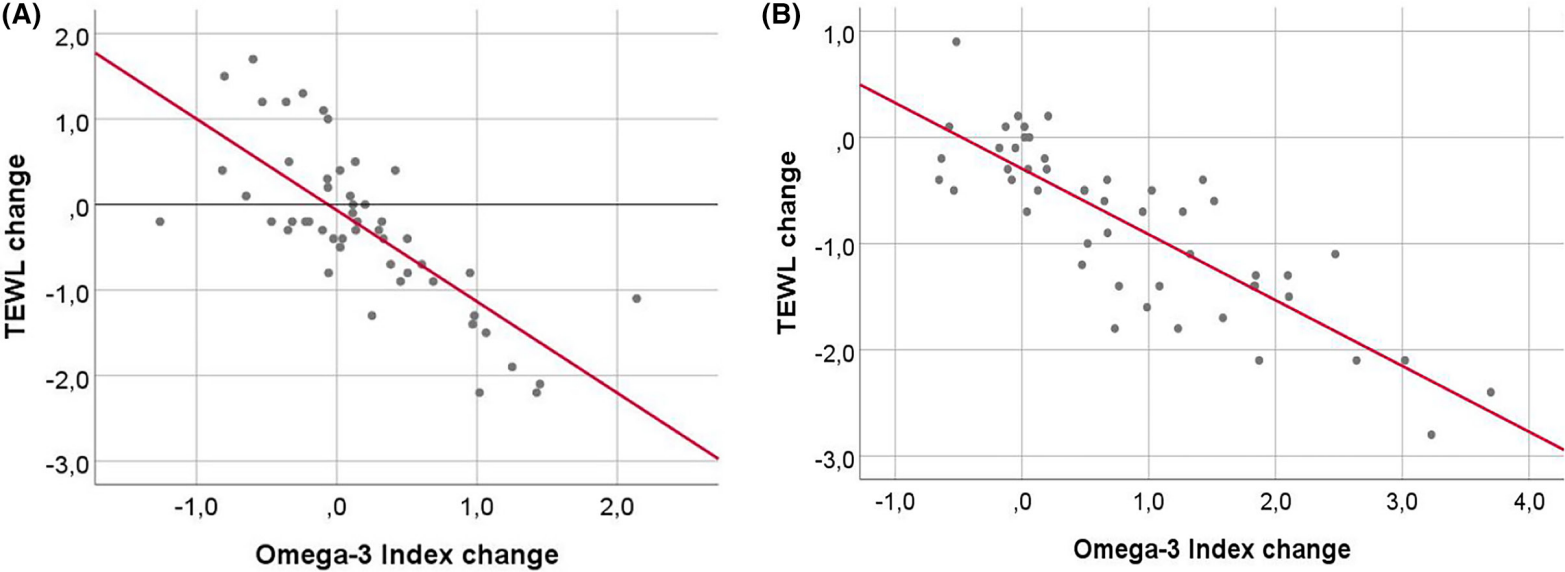
Changes in TEWL from baseline (0D) to trial end (84D) as a function of changes in Omega‐3 index across the same time period. (A) Data from Study 1 (1 g/day of krill oil/placebo). (B) Data from Study 2 (2 g/day of krill oil/placebo).

In study 2 (2 g/day), a significant time × supplement interaction effect on TEWL was observed (*p* < 0.001). Post hoc analysis showed that krill oil led to a more substantial TEWL decrease compared to placebo, with the krill oil group reducing TEWL from 14.25 ± 3.21 (mean ± SD) at baseline to 13.02 ± 2.76 by Day 84 (*p* < 0.0001; Cohen's *d* = 1.56). While the placebo group also showed a significant TEWL change from 12.30 ± 2.91 (mean ± SD) to 12.06 ± 3.10 (*p* = 0.015), an independent *t*‐test revealed a significantly greater reduction in the krill oil group (*t* = −6.13, *p* < 0.001; 95% CI = [−1.35, −0.64], Cohen's *d* = 1.74). Figure 2 displays the relationship between changes in omega‐3 index and TEWL in study 1 and study 2. In both studies, linear regression revealed a significant negative correlation between change of omega‐3 index and change of TEWL (study 1: *r* ^2^ = 0.56, *p* < 0.001, and study 2: *r* ^2^ = 0.70, *p* < 0.001). This suggests that the increases in the omega‐3 index resulting from krill oil supplementation were associated with the reductions in TEWL.

### 5.4 Hydration

In study 1, a significant time × supplement interaction effect on skin hydration was found (*p* = 0.012). Post hoc analyses revealed that the krill oil group showed greater increases in skin hydration compared to placebo, with a significant rise from 46.31 ± 6.61 (mean ± SD) to 48.31 ± 6.93 by Day 84 (*p* < 0.001; Cohen's *d* = 1.23; Table 3). There was also a significant change in hydration in the placebo group (*p* = 0.013), but the independent samples *t*‐test revealed that the krill oil group showed a more pronounced increase (*t* = 2.69, *p* = 0.01; 95% CI = [0.31, 2.09], Cohen's *d* = 0.76).

A significant time × supplement interaction effect on hydration was also found in study 2 (*p* < 0.001). Post hoc analyses revealed that the krill oil group showed greater increases in skin hydration compared to placebo, with scores rising from 47.52 ± 4.42 (mean ± SD) to 51.17 ± 4.51 by Day 84 (*p* < 0.0001; Cohen's *d* = 1.71; Table 3). This study also showed a significant change in hydration in the placebo group (*p* = 0.007), but the krill oil group showed a significantly more pronounced increase (*p* < 0.001; 95% CI = [1.81, 3.79], Cohen's *d* = 1.62). Linear regression found significant positive linear relationships between the change of omega‐3 index and change of hydration in both study 1 (*r* ^2^ = 0.41, *p* < 0.001) and study 2 (*r* ^2^ = 0.47, *p* < 0.001), suggesting that increases in the omega‐3 index due to krill oil supplementation was associated with increases in skin hydration in both studies.

### 5.5 Elasticity

In study 1, a significant time × Supplement interaction effect on skin elasticity was found (*p* < 0.001). Post hoc analyses revealed that the krill oil group showed greater increases in elasticity than placebo, with a significant rise from 0.788 ± 0.080 AU (mean ± SD) to 0.803 ± 0.079 by Day 84 (*p* < 0.001; Cohen's *d* = 1.19), (Table 3). There was also a significant change in elasticity in the placebo group (*p* = 0.004), but the independent samples *t*‐test showed that the krill oil group had a more pronounced increase (*t* = 4.69, *p* < 0.001; 95% CI = [0.0068, 0.0171], Cohen's *d* = 1.44).

A significant time × supplement interaction effect on elasticity was also found in study 2 (*p* < 0.001). Post hoc analyses revealed that the krill oil group showed a stronger improvement in elasticity compared to placebo, with scores rising from 0.830 ± 0.075 (mean ± SD) to 0.858 ± 0.074 by Day 84 (*p* < 0.0001; Cohen's *d* = 2.12) (Table 3). A significant change in elasticity was also evident in the placebo group (*p* = 0.002), but the independent samples *t*‐test revealed that the krill oil group had a more pronounced increase (*t* = 8.92, *p* < 0.001; 95% CI = [0.0179, 0.0284], Cohen's *d* = 2.61). Linear regression found a significant positive linear relationship between change of omega‐3 index and change of skin elasticity in both studies (study 1: *r* ^2^ = 0.56, *p* < 0.001 and study 2: *r* ^2^ = 0.78, *p* < 0.001) suggesting that the increases in the omega‐3 index due to Superba krill oil supplementation was associated with increases in skin elasticity.

## 6 DISCUSSION

The results from these two pilot studies in healthy volunteers suggest that krill oil supplementation may have the ability to improve the skin's capability to retain water in addition to improving skin hydration and elasticity. Daily oral supplementation with 1 and 2 g of krill oil showed significant and dose‐dependent improvements in skin TEWL, hydration, and elasticity compared to placebo. In addition, significant associations were found between these changes and the change in the omega‐3 index following krill oil supplementation. It is intriguing that the intervention resulted in advantageous alterations among a population initially categorized with healthy TEWL levels, ranging between 12.30 and 14.47 g/m^2^/h. The probability of the observed changes in the skin‐related outcomes being attributed to the krill oil supplementation is increased by the consistent and stronger response across all three outcomes at the 2 g/day compared to the 1 g/day dosage, and by the linear relationships observed between the changes in each skin outcome and the omega‐3 index. Indeed, the 1 g/day dose led to a mean percentage decrease of 4.42% whereas the 2 g/day dose led to an 8.63% decrease in TEWL over the 12‐week study period.

Maintaining optimal skin TEWL and hydration are indicative of a robust barrier function, which is important to safeguard against external irritants and for supporting processes such as cell turnover and repair. Skin elasticity, influenced by collagen and elastin, is vital for a youthful appearance and preventing wrinkles.^41^ A recent study with a similar design assessed the effects of 2 g/d krill oil or placebo supplementation in a Japanese population.^32^ At baseline, this population had a higher omega‐3 index (7.5%) and a lower baseline TEWL (10 g/h/m^2^) compared to our study population (5.5% and 13 g/h/m^2^, respectively), which aligns with the observed negative association between omga‐3 index and TEWL. The higher baseline omega‐3 index in the Japanese study is probably due to the higher habitual fish intake in Japan compared to the UK. Nevertheless, supplementation with 2 g/d krill oil for 12 weeks led to a mean change of 1.7% in the omega‐3 index which is comparable to our results, and TEWL decreased significantly compared to placebo in the Japanese study by a mean of −1.3 g/h/m^2^ similar to the −1.23 g/h/m^2^ observed with the same dose in our study.^32^

Two pre‐clinical studies in mice can shred light over the potential mechanism of action for the effects of krill oil supplementation on skin health. Chun et al. found that an 8‐week long krill oil intervention in 80 ICR mice increased skin ceramide, water and hyaluronan levels in a dose‐dependent manner by increasing their expression of genes coding for hyaluronic acid and collagen synthesis compared to controls.^33^ Another study by Kim et al., supplemented 60 hairless mice with krill oil or control for 15 weeks and exposed them to UVB radiation. The UVB radiation induced skin wrinkles in the mice, but krill oil suppressed the formation in terms of length and depth by increasing skin collagen and hyaluronic acid contents, also through increasing skin mRNA expression of hyaluronic acid‐ and collagen‐related genes (Has1, Has2, Has3, COL1A1, and COL1A2) compared to control. In addition, krill oil mitigated UVB induced inflammation by reducing inflammatory cytokines and reducing UVB induced oxidative stress compared to control.^34^ Collagen, a fibrous protein, plays a crucial role in reducing TEWL and enhancing hydration by providing structural support to the skin that helps to create a more intact barrier, maintaining its firmness and elasticity, thereby reducing the amount of water that can escape from the skin.^42^

On the other hand, hyaluronic acid is a powerful humectant, meaning it attracts and holds onto water. It can hold up to 1000 times its weight in water, which helps to keep the skin hydrated by drawing moisture from the environment and deeper layers of the skin.^43^ By maintaining high hydration levels, hyaluronic acid helps to strengthen the skin's natural barrier, further reducing TEWL and protecting against dehydration.^12^

Together, collagen and hyaluronic acid improve the skin's ability to retain moisture and maintain its barrier function, effectively reducing TEWL and enhancing overall skin hydration.^35^, ^36^ Their increased expression after krill oil supplementation in animal models might therefore provide plausible mechanistic explanations for the effects seen with krill oil in the present studies as well as the Japanese study.

Krill oil may also influence the amount of ceramides and the ceramide fatty acid composition in the epidermis.^22^ Having an adequate level of ceramides is crucial for proper functioning of the stratum corneum.^44^ Studies have shown that EPA, but not DHA, was able to alter the production of ceramides of ex vivo skin explants^21^ and ameliorate atopic dermatitis‐like symptoms in mice by restoring covalently bound ceramides in the stratum corneum.^45^ Since the fatty acid composition of krill oil has an EPA to DHA ratio of about 2:1, this might be of relevance in its ability to affect cutaneous ceramides. Hence, future clinical trials might want to strengthen the results by analyzing skin ceramide concentrations and fatty acid composition, in addition to skin biopsies for gene expression, eicosanoid and ceramide metabolites, and expand the study population to include older individuals or those with atopic dermatitis or psoriasis. Since an interaction between the microbiome and skin barrier function has been suggested, a further interesting research avenue might be to investigate the effect of krill oil on the microbiome.^46^ In earlier preclinical investigations, the impact of krill oil on gut microbiota was identified, yet its correlation with skin health has not been established so far.^47^, ^48^, ^49^

The strengths of these two studies with different doses include the randomized, double‐blind, controlled, study design which reduces the risk of bias. The fact that two different doses were investigated with better effects seen in the higher dose group, in addition to the associations with the change in the omega‐3 index as a validated and objective biomarker for long‐term omega‐3 status and compliance,^38^ strengthens the likelihood that the observed effects are related to the krill oil supplementation. TEWL has been used as an endpoint in dermatology studies for claim support and efficacy testing for three decades, and the tewameter has proven excellent intra‐ and inter‐rater reliability.^50^ Limitations of the studies include the lack of a proper power calculation and collection of baseline characteristics of the participants (age, BMI, sex, background dietary intake, etc.) due to the pilot/dose‐finding nature of these trials. The relatively high dropout rate in both studies must also be considered a limitation and dropouts should have been invited to attend a withdrawal visit so that their data could be used in intention‐to‐treat analyses.

## 7 CONCLUSION

The results from these two studies in healthy volunteers suggest that krill oil supplementation may improve the skin's capability to retain water, in addition to improving its hydration and elasticity. Maintaining these functions are crucial for the skin to safeguard against external irritants, support cell turnover and repair, and to maintain a youthful appearance and preventing wrinkles. Daily oral supplementation with 1 and 2 g of krill oil showed significant and dose‐dependent improvements when compared to placebo. In addition, significant associations were found between these changes and the omega‐3 index. Given its high safety profile, relatively low cost, and ease of supplementation, krill oil supplementation is a reasonable intervention that may benefit people who wish to improve their skin barrier function and overall skin health through diet. The integration of microarrays, lipidomics, and microbiome analysis could serve as comprehensive analytical approaches to fully understand the diverse pathways influenced by krill oil and their promotion of skin health.

## CONFLICT OF INTEREST STATEMENT

KH and LB are employees of Aker BioMarine Human Ingredients AS that has provided the krill and placebo oil and funded the study.

## ETHICS STATEMENT

The studies were conducted according to the guidelines of the Declaration of Helsinki, and the protocols and informed consent were approved by the Institutional Review Board (IRB) of East Midlands Leicester Central Research Ethics Committee on 15.4.2015 and extended by amendment.

## Data Availability

The data that support the findings of this study are archived in a public repository and can be accessed via this link: https://akerbiomarine.box.com/s/xr44pzcr7s80fov1p4o3widghn3yrjpj.
